# Changes in sleep quality and sleep disturbances in the general population from before to during the COVID-19 lockdown: A systematic review and meta-analysis

**DOI:** 10.3389/fpsyt.2023.1166815

**Published:** 2023-04-13

**Authors:** Federica Limongi, Paola Siviero, Caterina Trevisan, Marianna Noale, Filippo Catalani, Chiara Ceolin, Silvia Conti, Elisa di Rosa, Elena Perdixi, Francesca Remelli, Federica Prinelli, Stefania Maggi

**Affiliations:** ^1^Aging Branch, Neuroscience Institute, National Research Council, Padova, Italy; ^2^Department of Medical Sciences, University of Ferrara, Ferrara, Italy; ^3^Geriatric Unit, Department of Medicine, University of Padova, Padova, Italy; ^4^Unit of Behavioral Neurology and Dementia Research Center, IRCCS Mondino Foundation, Pavia, Italy; ^5^Department of General Psychology, University of Padova, Padova, Italy; ^6^Epidemiology Unit, Institute of Biomedical Technologies, National Research Council, Milano, Italy

**Keywords:** sleep quality, sleep disturbances, sleep onset latency, sleep efficiency, insomnia, general population, COVID-19 lockdown, changes

## Abstract

**Introduction:**

This systematic review and meta-analysis aims to explore changes in sleep quality and sleep disturbances in the general population from before to during the COVID-19 lockdown.

**Methods:**

The protocol was registered in PROSPERO (CRD42021256378) and the PRISMA guidelines were followed. The major databases and gray literature were systematically searched from inception to 28/05/2021 to identify observational studies evaluating sleep changes in the general population during the lockdown with respect to the pre-lockdown period. A random effects meta-analysis was undertaken for studies reporting (a) the means of the Pittsburgh Sleep Quality Index (PSQI) global scores or the means of the sleep onset latency (SOL) times (minutes - min) before and during the lockdown, (b) the percentages of poor sleep quality before and during the lockdown, or (c) the percentages of changes in sleep quality. Subgroup analysis by risk of bias and measurement tool utilized was carried out. A narrative synthesis on sleep efficiency, sleep disturbances, insomnia and sleep medication consumption was also performed.

**Results:**

Sixty-three studies were included. A decline in sleep quality, reflected in a pooled increase in the PSQI global scores (standardized mean difference (SMD) = 0.26; 95% CI 0.17–0.34) and in SOL (SMD = 0.38 min; 95% CI 0.30–0.45) were found. The percentage of individuals with poor sleep quality increased during the lockdown (pooled relative risk 1.4; 95% CI 1.24–1.61). Moreover, 57.3% (95% CI 50.01–61.55) of the individuals reported a change in sleep quality; in 37.3% (95% CI 34.27–40.39) of these, it was a worsening. The studies included in the systematic review reported a decrease in sleep efficiency and an increase in sleep disturbances, insomnia, and in sleep medication consumption.

**Discussion:**

Timely interventions are warranted in view of the decline in sleep quality and the increase in sleep disturbances uncovered and their potentially negative impact on health. Further research and in particular longitudinal studies using validated instruments examining the long-term impact of the lockdown on sleep variables is needed.

**Systematic review registration:**

https://www.crd.york.ac.uk/prospero/display_record.php?ID=CRD42021256378, identifier CRD42021256378.

## Introduction

Alarmed by its severity, transmissibility, rising levels of contagion, and the strain on healthcare systems, on March 11, 2020, the World Health Organization (WHO) declared the Coronavirus disease 2019 (COVID-19) outbreak, a global pandemic (1). In the absence of vaccines or pharmaceutical treatment, non-pharmaceutical interventions (NPIs) were implemented in most countries of the world to limit the diffusion of the virus and to mitigate the burden on health systems. The NPIs included strict hand hygiene and the use of face masks and more restrictive measures such as isolation, quarantine, social distancing, curfews, travel bans, remote working, school closures, and full or partial lockdowns (2). A growing body of evidence has, however, shown that these restrictive measures have adversely affected people’s mental health and well-being. For instance, in several studies more stringent NPIs have been associated with higher anxiety and depressive symptoms (3), a decrease in mental well-being (4, 5) as well as to an increase in psychological distress (6).

The negative influence of the COVID-19 pandemic and restrictive measures on mental wellbeing translated also in a significant impact on sleep health. Several studies have in fact described an increase in sleep duration, delayed sleep timing, and a reduction in sleep variability (a feature of social jet lag) during the pandemic with only a gradual return of some of these parameters to pre-pandemic levels when those measures were revoked or mitigated (7, 8). Not all the data regarding the effects of restrictive measures on sleep disturbances are, however, congruent. If on the one hand, the prevalence of sleep disturbances appeared to be higher during the lockdown with respect to non-lockdown periods (9, 10), two systematic reviews and meta-analyses have reported an inverse association between the severity of restrictive measures and the prevalence of sleep disturbances (11, 12). Nutrition, physical activity and sleep are almost unanimously considered the three pillars of health. In particular, in reference to sleep, it is known that quantitative and/or qualitative sleep disturbances can have short- and long-term consequences such as worse cognitive performance, mood disorders, worse quality of life, hypertension, diabetes, weight gain/obesity, cardiovascular and cerebrovascular diseases, and increased mortality (13, 14). Several systematic reviews and meta-analyses evaluating the impact of the COVID-19 pandemic on sleep disturbances in different populations, including the general one, reported that sleep disturbances during the COVID-19 pandemic were common, although the general population seemed to be the least affected (9–12, 15, 16). Those works did not evaluate changes in sleep quality and sleep disturbances during the lockdown with respect to pre-lockdown levels nor did they focus on the lockdown, which, by any definition, was an extraordinary measure taken by authorities constraining practically all citizens living in that country to make radical changes in their daily routine. Even if three systematic reviews and meta-analyses evaluated changes in mental health outcomes, including sleep disturbances, during the pandemic (17, 18) or lockdown (19) with respect to before, only a very few studies examining sleep disorders were included. The current systematic review and meta-analysis aimed to present an overview and synthesis of changes in sleep quality and sleep disturbances during the lockdown in the general population with respect to the pre-lockdown levels, and more specifically to delineate the changes in several different sleep outcomes such as sleep onset latency, sleep efficiency and insomnia symptoms.

## Methods

### Protocol and registration

This systematic review was conducted in accordance with the Preferred Reporting Items for Systematic Reviews and Meta-Analyses (PRISMA) guidelines (20) and registered in the International Prospective Register of Systematic Reviews (PROSPERO, protocol ID: CRD42021256378).

### Search strategy

Four electronic academic databases (PubMed, Cochrane Library, Ebsco, and Web of Science -WOS), a preprint server (MedRxiv), and a gray literature database (OpenGrey) were searched systematically from inception to 28/05/2021, corresponding to the end of the 1st year of the pandemic, using the search terms listed in the Supplementary Table 1.

All the references were downloaded in Zotero, a citation manager software used for all the steps of the studies’ selection process, from downloading and removing duplicates, to the title-abstract and full-text screenings, which were performed independently by two researchers (EP, FR). The reference lists of the relevant systematic reviews and of the articles identified were checked for references that might lead to additional studies. To mediate any disputes, the final decision regarding a study’s eligibility was made together with the senior authors (SM, CT, and FP).

### Selection criteria

The inclusion and exclusion criteria used, in accordance with the PICOS (Population, Intervention, Comparison, Outcome, and Study design) description, are outlined below.

#### Inclusion criteria

Population: general adult population (≥18 years) and studies whose samples were mainly composed of adults with adolescents making up at most 30%.

Intervention(s)/exposure: COVID-19 lockdown.

Outcomes: changes in sleep characteristics assessed by self-report validated instruments [e.g., Pittsburgh Sleep Quality Index – PSQI (21), Insomnia Severity Index - ISI (22)], self-report researcher-developed tools or device-based measures.

The PSQI is a self-report questionnaire that assesses global sleep quality relative to the previous 30 days’ time. The global score ranges from 0 to 21, with a cut-off >5 indicating “poor sleep quality.” The questionnaire also evaluates seven sleep components: subjective sleep quality, sleep latency, sleep duration, sleep efficiency, sleep disturbances, sleep medication use, and daytime dysfunction (21). The ISI is a 7-item self-report questionnaire that assesses the nature, severity, and impact of insomnia on a scale from 0 to 28. The scores have been classified as: 0–7 = “no insomnia,” 8–14 = “subthreshold or mild insomnia,” 15–21 = “clinical insomnia or moderate insomnia,” and 22–28 = “severe clinical insomnia” (22).

The current study has focused on the following sleep characteristics: sleep quality, sleep onset latency (the length of time, in minutes, it takes to transition from wake to sleep), sleep efficiency (the ratio of total sleep time to time in bed) (23), sleep disturbances, insomnia, insomnia types (sleep onset insomnia, sleep maintenance insomnia, early morning awakening insomnia), and sleep medication consumption.

Study design: original observational cross-sectional, prospective or retrospective cohort studies.

#### Exclusion criteria

(1) Studies in languages other than English, Italian or Spanish;

(2) Studies evaluating changes in subjects with specific diseases (e.g., obesity, diabetes, neuromuscular disease, cancer, osteoarthritis, and dementia) and in specific groups not representing the general population (e.g., professional athletes, health-care workers).

### Data extraction

The data were extracted by two authors (EP, FR) using a pre-designed spreadsheet: the first author’s name, year of publication, country, study design, outcome, the sample size, the measurement tool utilized, the percentage of female participants, the participants’ ages (mean, median, or interval), the assessment period, and the main study results on sleep health changes. The corresponding author was contacted whenever a study appeared incomplete or in case any clarification on the presented data was needed.

### Risk of bias assessment

The risk of bias was assessed by two independent authors (FL, PS) using the Newcastle Ottawa Scale (NOS) for longitudinal (24) and cross-sectional studies (25). The NOS assesses selection, comparability, and outcome by assigning a congruent number of stars: cross-sectional studies can achieve a score from 0 to 10 stars and the longitudinal ones a score from 0 to 9, with higher scores corresponding to a lower risk of bias. Studies whose NOS <5 are identified as having a low quality and a high risk of bias (26). A third author (MN) was involved in resolving any discrepancies.

### Data synthesis

A meta-analysis was performed for data regarding sufficiently homogenous outcomes in terms of statistical and methodological characteristics. A narrative synthesis approach was used for those studies not included in the meta-analysis.

#### Meta-analysis

A random-effects meta-analysis was carried out for sleep quality and sleep onset latency, using the DerSimonian and Laird method, with studies weighted according to the inverse of the standard error, using MedCalc Statistical Software version 20.118 (27).

Studies reporting the following data were included in the meta-analysis:

• the means of the PSQI global scores or the means of the sleep onset latency times (minutes - min) before and during the lockdown;
• the percentages of poor sleep quality (researcher-developed questions or PSQI global score >5) before and during the lockdown;
• the percentages of changes in the individuals who improved or worsened, or maintained the same sleep quality during the lockdown with respect to pre-lockdown levels.

For each type of data, the effect was expressed as standardized mean difference (SMD) (Cohen’s rule of thumb for the interpretation of the total SMD suggested that a value of 0.2 indicates a small effect, a value of 0.5 indicates a medium effect and a value of 0.8 or larger indicates a large effect), relative risks or proportions. The between-study heterogeneity was analyzed using *I*^2^ statistic: a value of 0% indicates no observed heterogeneity, and higher values show increasing heterogeneity (28). The publication bias was assessed with Egger’s test (29).

#### Subgroup analysis

Given the different ways that the data were synthesized, whenever possible, additional stratified meta-analyses by risk of bias (NOS <5 vs. NOS ≥5) and measurement tool utilized (self-reported validated vs researcher-developed instruments) were conducted. *T*-test and Chi-squared test were used to compare subgroups.

## Results

**FIGURE 1:**
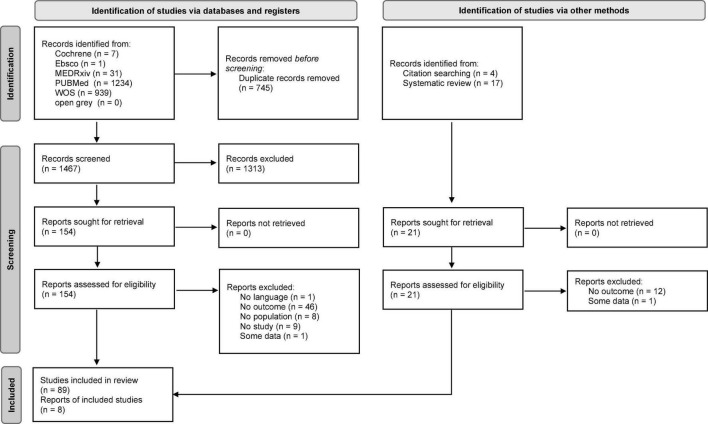
PRISMA flow diagram showing the process of study selection.

The initial search yielded 2,212 results. After removing 745 duplicates, 1,467 articles were retained and screened based on the title and abstract. Of these, 154 together with other 21 studies identified *via* citation searches and systematic reviews were selected for full-text screening. Ninety-seven articles were selected for inclusion when the full texts were assessed. Of these, 63 contained data on outcomes pertinent to this study (30–92): 49 on sleep quality (30–33, 36–51, 53–55, 57, 60–63, 65, 67–69, 71–74, 76, 80–89, 91, 92), 16 on sleep onset latency (43, 48–50, 54, 57, 61, 63, 71, 73, 78, 83, 85, 88, 90, 92), 9 on sleep efficiency (43, 49, 63, 73, 77, 78, 83, 85, 88), 10 on sleep disturbances (35, 45, 46, 52, 58, 63, 70, 73, 83, 88), 13 on insomnia and insomnia symptom types (5 on insomnia, 8 on sleep onset insomnia, 8 on sleep maintenance insomnia, 5 on early morning awakening insomnia) (34, 45, 46, 49, 54, 56, 59, 61, 64, 66, 71, 73, 75), and 11 on sleep medication consumption (35, 43, 49, 50, 56, 61, 63, 70, 79, 83, 88). The complete PRISMA flow diagram is shown in Figure 1. The characteristics of the studies included are shown in Table 1. The studies were conducted in Argentina (*N* = 1), Australia (*N* = 2), Brazil (*N* = 1), China (*N* = 5), Cyprus (*N* = 1), France (*N* = 4), Germany (*N* = 3), India (*N* = 5), Italy (*N* = 8), Libya (*N* = 1), Multi-country (*N* = 9), Romania (*N* = 1), Russia (*N* = 1), Saudi Arabia (*N* = 1), Singapore (*N* = 1), Spain (*N* = 7), UK (*N* = 6), United Arab Emirates (*N* = 1), and USA (*N* = 5). Due to pandemic restrictions, most of the studies collected data through online surveys. Sixty-one studies used self-reported instruments (30–76, 79–92) and 2 device-based (DB) ones (77, 78). Of the former, 20 used validated instruments (32, 33, 42, 43, 47, 51, 54, 61, 63, 65, 66, 68, 69, 71–73, 84, 85, 88, 90) (17 PSQI), and 41 used researcher-developed ones (31, 34–41, 44–46, 48–50, 52, 53, 55–60, 62, 64, 67, 70, 74–76, 79–83, 86, 87, 89, 91, 92). With regard to the researcher-developed instruments, some studies utilized selected items of validated tools, but in the majority of cases the investigators asked the participants to evaluate if they had noted a change (either an improvement, worsening or no change) in the sleep outcome being examined during the lockdown with respect to pre-lockdown status. Forty-eight of the studies were cross-sectional (30, 31, 33–50, 52, 53, 57, 58, 60–64, 66, 67, 69–71, 73–75, 79–83, 86–90, 92) and 15 were longitudinal (32, 51, 54–56, 59, 65, 68, 72, 76–78, 84, 85, 91). The mean NOS score for the cross-sectional studies was 4.1 (SD = 1.3; range 1-7); it was 5.1 (SD = 1.0; range 3-8) for the longitudinal studies (see Supplementary Table 2 for full scoring information). Out of the total number of studies, 49.2% (31 studies) were of high quality.

**TABLE 1:** Descriptive characteristics of included studies.

References	Country	Study design	Assessment period	Population	N	Age	Data collection/Type of recruitment	Measurement tool	Risk of bias score	Q	SOL	SE	SDIST	IN	SOI	SMI	EMA	MED
Aguiar et al. (30)	Brazil	cross-sectional	August 17–31, 2020	University students	527; F 81%	24 ± 5.6 y	Online questionnaire/snowball sampling	Researcher- developed questions	5	1								
Allen et al. (31)	UK	cross-sectional	April 15–June 8, 2020	General population	200; F 86%	18–62 y; 24.7 ± 7.2 y	Online questionnaire/convenience sampling	Researcher- developed questions	4	1								
Al-Musharaf et al. (32)	Saudi Arabia	longitudinal	B: February–April, 2019; D: April–May, 2020	University students	297; F 100%	19–30 y; 20.7 ± 1.4 y	Telephonic interview/NR	PSQI	5	1								
Ammar et al. (33)	Multi-country: Western Asia/North Africa/Europe/Americas	cross-sectional	April 6–11, 2020	General population	1047; F 53.8%	≥18 y	Online survey/convenience sampling	PSQI	3	1								
Bacaro et al. (34)	Italy	cross-sectional	April 1–May 4, 2020	General population	1989; F 76.2%	38.4 ± 12.8 y	Online survey/convenience sampling	Researcher- developed questions	5					1				
Beck et al. (35)	France	cross-sectional	March 31–April 2, 2020	General population	1500; F 52%	≥18 y	Online survey/random sampling	Researcher- developed questions	6				1					1
Ben Salah et al. (36)	Multi-country: USA/Germany/India/Tunisia	cross-sectional	March 31–May 15, 2020	General population	3816; F 70.5%	≥18 y; 38.6 ± 14 y	Online survey/convenience sampling	Researcher- developed questions	3	1								
Bigalke et al. (37)	USA	cross-sectional	April 25–May 18, 2020	General population	103; F 59%	Media 38y	Online survey/convenience sampling	Researcher- developed questions	5	1								
Blume et al. (38)	Multi-country: Austria/Germany/Switzerland	cross-sectional	March 23–April 26, 2020	General population	435; F 75.2%	≥18 y; median 26–35 y	Online survey/convenience sampling	Researcher- developed questions	4	1								
Brindal et al. (39)	Australia	cross-sectional	May 2020	General population	3745; F 85.7%	56.4 ± 12.6 y	Online survey/convenience sampling	Researcher- developed questions	2	1								
Buoite Stella et al. (40)	Italy	cross-sectional	March 23–29, 2020	General population	400; F 69%	35 ± 15 y	Online survey/convenience sampling	Researcher- developed questions	6	1								
Cancello et al. (41)	Italy	cross-sectional	April 15–May 4, 2020	General population	490; F 84%	≥18 y	Online survey/convenience sampling	Researcher- developed questions	2	1								
Cellini et al. (42)	Italy	cross-sectional	March 24–28, 2020	Students, workers	1310; F 67.2%	23.91 ± 3.6 y	Online survey/convenience sampling	PSQI	5	1								
Cellini et al. (43)	Multi-country: Italy/Belgium	cross-sectional	April 1–May 19, 2020	General population	1662 Italians, 650 Belgian; F 72.19%, 78.3%	34.1 ± 13.6 y; 43.0 ± 16.8 y	Online survey/convenience sampling	PSQI	5	1	1	1						1
Celorio-Sardà et al. (44)	Spain	cross-sectional	May 22–July 3, 2020	Students, workers	321; F 79.8%	≥18 y	Online survey/convenience sampling	Researcher- developed questions	3	1								
Cheikh Ismail et al. (45)	Multi-country: MENA region.	cross-sectional	April 15–29, 2020	General population	2970; F 71⋅6%	≥18 y	Online survey/convenience and snowball sampling	Researcher- developed questions	5	1			1		1	1	1	
Cheikh Ismail et al. (46)	United Arab Emirates	cross-sectional	April and May 2020	General population	1012; F 75.9%	≥18 y	Online survey/snowball sampling	Researcher- developed questions	5	1			1		1	1	1	
Chopra et al. (47)	India	cross-sectional	August 15–30, 2020	General population	995; F 41.4%	33.33 ± 14.5 y; range 18–85 y	Online and telephonic survey/quota sampling	Validated questionnaire	5	1								
Chouchou et al. (48)	France	cross-sectional	35th–45th days of lookdown	General population	400; F 58.3%	≥18 y; 29.8 ± 11.5 y	Online survey/convenience sampling	Researcher- developed questions (PSQI, selected items)	4	1	1							
Diz-Ferreira et al. (49)	Spain	cross-sectional	March 30–April 12, 2020	General population	451; F 73.4%	≥18 y	Paper and online survey/convenience sampling	Researcher- developed questions (OSQ adapted)	7	1	1	1		1				1
Elhadi et al. (50)	Libya	cross-sectional	July 18–August 23, 2020	General population	10296; F 76.6%	28.9 ± 8.5 y	Online survey/convenience sampling	Researcher- developed questions	3	1	1							1
Evans et al. (51)	UK	longitudinal	B: October 2019; D: May 1–30, 2020	Students	254; F 86.2%	18–31 y; 19.76 ± 1.28 y	Paper and online survey/NR	PSQI	4	1								
Fernandez-Ballesteros and Sánchez-Izquierdo (52)	India	cross-sectional	April 1–May 5, 2020	General population	315; F 47.9%	60–93 y	Online survey/convenience sampling	Researcher- developed questions	2				1					
Flanagan et al. (53)	Multi-country: USA/Australia/Canada/Ireland/UK	cross-sectional	April 3–May 3, 2020	General population	7753; F 80.0%	≥18 y; 51.2 ± 0.17 y	Online survey/convenience sampling	Researcher- developed questions	5	1								
Gao and Scullin (54)	USA	longitudinal	B: February 17, 2020; D: March 25–27, 2020	General population	699 (B:199; D: 500; B/D: 86); F 44.78%	38.04 ± 11.65 y	Online survey/convenience sampling	PSQI + researcher developed questions (SOL, SMI)	5	1	1					1		
García-Esquinas et al. (55)	Spain	longitudinal	B: 2019; D: April 27–June 22, 2020	General population	3041; F 57.7%	≥65 y; mean 74.5 y	Telephonic interview/cohort	Researcher- developed questions	5	1								
Ge et al. (56)	China	longitudinal	B: January 1–December 31, 2019; D: February 1–March 8, 2020	General population	2061; F 32.6%	≥18 y; 27.21 ± 7.16 y	Online survey/snowball sampling	Researcher- developed questions (PSQI, selected items)	3						1			1
Gupta et al. (57)	India	cross-sectional	April 28–May 10, 2020	General population	958; F 41.2%	37.32 ± 13.09 y	Online survey/snowball sampling	Researcher- developed questions	5	1	1							
Hetkamp et al. (58)	Germany	cross-sectional	March 10–April 30, 2020	General population	16245; F 70.8%	≥18 y	Online survey/convenience sampling	Researcher- developed questions (PHQ-9, single item)	3				1					
Hisler and Twenge (59)	USA	longitudinal	B: 2018; D: April 27, 2020	General population	B: 19433, D: 2059; F 51.8%, 50.7%	≥18 y; B: 42.84 ± 14.84 y; D: 43.35 ± 14.88 y	Online survey/probability sampling	Researcher- developed questions	5						1	1		
Ingram et al. (60)	UK (Scotland)	cross-sectional	First lookdown	General population	399; F 56.4%	18–72 y; 32.4 ± 11.4 y	Online survey/convenience sampling	Researcher- developed questions	3	1								
Innocenti et al. (61)	Italy	cross-sectional	First lookdown	General population	1035; F 82.9%	≥18 y; 30–50 y	Online survey/convenience sampling	PSQI	1	1	1					1		1
Knell et al. (62)	USA	cross-sectional	April 15–May 5, 2020	General population	1809; F 67.4%	35–49 y;	Online survey/convenience sampling	Researcher- developed questions	3	1								
Kolokotroni et al. (63)	Cyprus	cross-sectional	April 10–May 12, 2020	General population	745; F 73.8%	≥18 y; median 39 (IQR 13) y; range 18–76 y	Online survey/convenience sampling	PSQI	4	1	1	1	1					1
Kontsevaya et al. (64)	Russia	cross-sectional	April 26–June 6, 2020	General population	2432; F 83%	≥18 y; 37.6 ± 13.4 y	Online survey/convenience sampling	Researcher- developed questions	6						1		1	
Leone et al. (65)	Argentina	longitudinal	B: February and May 2018 and 2019/February 2020; D: April 2020	General population	1021; F 69.64%	13–74 y; 37.4 ± 13.21 y	Online survey/convenience sampling	PSQI	6	1								
Lin et al. (66)	China	cross-sectional	February 5–23, 2020	General population	5641; F70.1%	≥18 y; 37.6 y	Online questionnaire/snowball sampling	ISI	5					1	1	1	1	
López-Moreno et al. (67)	Spain	cross-sectional	May 28–June 21, 2020	General population	675; F 30.1%	≥18 y; 39.1 ± 12.9 y; range 18–85 y	Online survey/snowball sampling	Researcher- developed questions	3	1								
Maher et al. (68)	USA	longitudinal	B: January 21–March 11, 2020; D: April 17–May 5, 2020	Students	107; F 66%	21.7 ± 2.6 y; 18–34	Online survey/convenience sampling	PSQI	4	1								
Majumdar et al. (69)	India	cross-sectional	April 14–May 2, 2020	University students, workers	325 students, 203 workers; F 60.9%, 18.2%	33.1 ± 7.11 y; 22.1 ± 1.66 y	Online survey/convenience sampling	ESS	2	1								
Mandelkorn et al. (70)	Multi-country: Multinations/USA	cross-sectional	March 26–April 26, 2020	General population	2562 study 1, 971 study 2; F 68%, 52.8%	45.18 ± 14.46 y; 40.36 ± 13.61 y	Online survey/convenience sampling	Researcher- developed questions (PSQI, ISI, SST: selected items)	4				1					1
Marelli et al. (71)	Italy	cross-sectional	March 24–May 2, 2020	University students, workers	400; F 75.8%	22.84 ± 2.68 y	Online survey/convenience sampling	PSQI, ISI	6	1	1			1	1	1	1	
Martinez-de-Quel et al. (72)	Spain	longitudinal	B: March 16–31, 2020; D: April 30–May 11, 2020	General population	161; F 37%	35.0 ± 11.2 y	Online survey/convenience sampling	PSQI	6	1								
Martínez-Lezaun et al. (73)	Spain	cross-sectional	After 20 days of lookdown	University students	102; F 80.4%	21.8 ± 2.97 y	Online questionnaire/convenience sampling quasi-experimental design	PSQI	3	1	1	1	1		1	1		
Micheletti Cremasco et al. (74)	Italy	cross-sectional	May 14–31, 2020	Students, workers	3666; F 73%	29.12 ± 12 y	Online survey/convenience sampling	Researcher- developed questions	5	1								
Mititelu (75)	Romania	cross-sectional	July 8–26, 2020	General population	805, F 19.7%	≥20 y	Online survey/convenience sampling	Researcher- developed questions	4					1				
Okely et al. (76)	UK (Scotland)	longitudinal	B: 2014–2017; D: May 27–June 8, 2020	General population	137; F 48.2%	mean 79 y	Online questionnaire/cohort	Researcher- developed questions (PSQI, adapted single item)	5	1								
Ong et al. (77)	Singapore	longitudinal	B: January 2–22, 2020; D: April 7–27, 2020	City-dwelling, workers	1824; F 51.64%	21–40 y; 30.94 ± 4.62 y	Device data/convenience sampling	DB (wrist-worn wearable technology)	8			1						
Pépin et al. (78)	France	longitudinal	B: February–March 16, 2020; D: March 17–May 11, 2020	General population	599; F 29%	Median 47 (IQR 36–59) y	Device data/convenience sampling	DB (Dream sleep-monitoring headband)	7		1	1						
Perez-Carbonell et al. (79)	UK	cross-sectional	May 12–June 2, 2020	General population	843; F 67.4%	≥18 y; median 52 (IQR 40–63) y	Online survey/convenience sampling	Researcher- developed questions	2									1
Robinson et al. (80)	UK	cross-sectional	April 28–May 22, 2020	General population	2002; F 61.7%	≥18 y; 34.74 ± 12.3 y	Online survey/convenience sampling	Researcher- developed questions	4	1								
Rossinot et al. (81)	France	cross-sectional	April 23– May 7, 2020	General population	1454; F 63.5%	24–65 y;	Online survey/convenience sampling	Researcher- developed questions	5	1								
Saalwirth and Leipold (82)	Germany	cross-sectional	April 1–19, 2020	General population	665; F 53.8%	18–73 y; 36 ± 14 y	Online questionnaire/convenience sampling	Researcher- developed questions (PSQI, adapted and selected items)	4	1								
Salehinejad et al. (83)	Germany	cross-sectional	April 20–28, 2020	General population	160; F 85.6%	18–60 y; 25.79 ± 7.31 y	Online survey/convenience sampling	Researcher- developed questions (PSQI, item 2)	4	1	1	1	1					1
Sañudo et al. (84)	Spain	longitudinal	B: February 2020; D: March 24–April 3, 2020	General population	20; F 45%	22.6 ± 3.4 y	Online questionnaire/convenience sampling	PSQI	4	1								
Sella et al. (85)	Italy	longitudinal	B: End 2017- mid 2018; D: April 27–May 4, 2020	General population	17 young, 21 older; F 52.9%, 52.4%	Young 18–35; older 65–90	Telephonic interview/convenience sampling	PSQI	5	1	1	1						
Sinha et al. (86)	India	cross-sectional	April 1–May 6, 2020	General population	1511; F 50.9%	≥18 y; 18–80 y	Online survey/convenience sampling	Researcher- developed questions	4	1								
Stanton et al. (87)	Australia	cross-sectional	April 9–9, 2020	General population	1491; F 67%	50.5 ± 14.9 y	Online survey/convenience sampling	Researcher- developed questions	4	1								
Trabelsi et al. (88)	Multi-country: Western Asia/North Africa/Europe/Americas	cross-sectional	April 6–June 28, 2020	General population	517; F 52.2%	≥56 y; 63.2 ± y	Online survey/convenience sampling	PSQI	5	1	1	1	1					1
Trakada et al. (89)	Multi-country: Greece/Switzerland/Austria/Germany/France/Brazil	cross-sectional	March 25–April 6, 2020 (Europe); June 10–14, 2020 (Brazil)	General population, health professionals	1908; F 68.9%	42.6 ± 12.7 y	Online questionnaire/convenience sampling	Researcher- developed questions	5	1								
Wang et al. (90)	China	cross-sectional	March 23–April 26, 2020	General population	2289; F 48.6%	27.5 ± 12.0 y; range 18–81 y	Online survey/convenience sampling	PSQI	4		1							
Zheng et al. (91)	China	longitudinal	B: 2019; D: April 15–26, 2020	General population	631 (B/D: 70); F 61.2%	18–35 y; 21.1 ± 2.9 y	Online survey/convenience sampling	Researcher- developed questions	5	1								
Zhu et al. (92)	China	cross-sectional	March 29–April 5, 2020	General population	889; F 61%	16–70 y; 31.8 ± 11.4 y	Online questionnaire/convenience sampling	Researcher- developed questions	3	1	1							

B, before the lockdown; D, during the lockdown; F, female; NR, not reported; PSQI, Pittsburgh Sleep Quality Index; ISI, Insomnia Severity Index; ESS, Epworth Sleepiness Scale; OSQ, Oviedo Sleep Questionnaire; PHQ-9, Patient-Health-Questionnaire-9; SST, Sleep Satisfaction Tool; Q, sleep quality; SOL, sleep onset latency; SE, sleep efficiency; SDIST, sleep disturbances; IN, insomnia: SOI, sleep onset insomnia; SMI, sleep maintenance insomnia; EMA, early morning awakening insomnia; MED, sleep medication consumption; MENA, Middle East and North Africa (Algeria, Bahrain, Egypt, Iraq, Jordan, Kuwait, Lebanon, Libya, Morocco, Oman, Palestine, Qatar, Saudi Arabia, Sudan, Syria, Tunisia, United Arab Emirates, and Yemen).

### Sleep quality

Forty-six out of 49 studies examining sleep quality were included in the meta-analysis: 23 reported data regarding both before and during the COVID-19 lockdown (32, 33, 40, 42, 43, 45–48, 51, 54, 55, 61, 63, 68, 71–73, 76, 83–85, 88), 24 reported percentage changes during the lockdown with respect to pre-lockdown levels (30, 31, 36, 37, 39, 41, 44, 50, 53, 54, 57, 60, 62, 65, 67, 74, 80–82, 86, 87, 89, 91, 92); the remaining three were only narratively described (38, 49, 69).

#### Meta-analytic changes in sleep quality

##### Means pre- and during-lockdown

**FIGURE 2:**
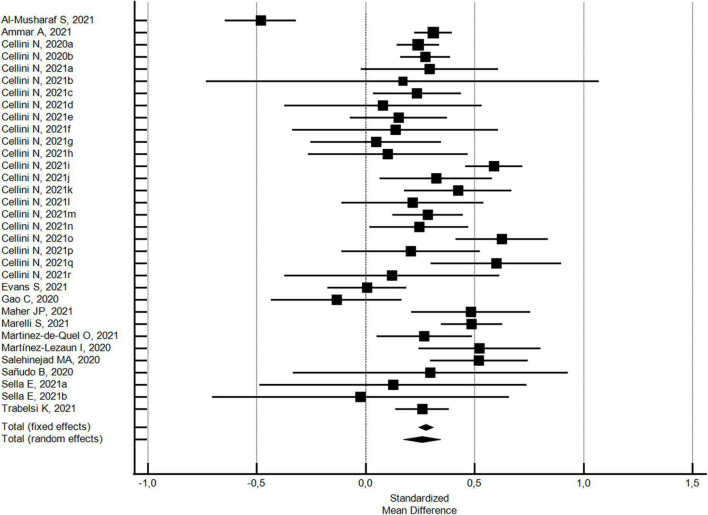
Forest plot showing pooled changes in sleep quality PSQI global score from before to during the COVID-19 lockdown. Cellini N, 2020a: Student; Cellini N, 2020b: Worker; Cellini N, 2021a: Belgian Students, Female; Cellini N, 2021b: Belgian Students, Male; Cellini N, 2021c: Belgian Regular workers, Female; Cellini N, 2021d: Belgian Regular workers, Male; Cellini N, 2021e: Belgian Remote workers, Female; Cellini N, 2021f: Belgian Remote workers, Male; Cellini N, 2021g: Belgian Unemployed/retired, Female; Cellini N, 2021h: Belgian Unemployed/retired, Male; Cellini N, 2021i: Italian Students, Female; Cellini N, 2021j: Italian Students, Male; Cellini N, 2021k: Italian Regular workers, Female; Cellini N, 2021l: Italian Regular workers, Male; Cellini N, 2021m: Italian Remote workers, Female; Cellini N, 2021n: Italian Remote workers, Male; Cellini N, 2021o:Italian Stop working, Female; Cellini N, 2021p: Italian Stop working, Male; Cellini N, 2021q: Italian Unemployed/retired, Female; Cellini N, 2021r: Italian Unemployed/retired, Male; Sella E, 2021a: Older; Sella E, 2021b: Young. Error bars = 95% confidence interval; Square boxes = individual study point estimates; and Diamond box = Pooled point estimates.

The changes in the PSQI global scores were evaluated considering 33 outcomes reported in 14 studies (32, 33, 42, 43, 51, 54, 56, 71–73, 83–85, 88). With regard to the PSQI global score (see Figure 2), there was a significant increase (SMD = 0.26; 95% CI 0.17–0.34; *I*^2^ = 80.6%) corresponding to a worsening in sleep quality. The analysis did not show a significant publication bias (Egger’s *p* = 0.568).

In addition, with regard to 27 outcomes in 8 studies (32, 42, 43, 54, 71, 72, 85, 88) with a high NOS quality score ≥5, the significant increase in the PSQI global score was substantially confirmed (SMD = 0.24; 95% CI 0.13-0.34; *I*^2^ = 82.2%; not significant Egger’s publication bias). With regard to the six studies (33, 51, 68, 73, 83, 84) with a low NOS quality score <5, the significant increase in the PSQI global score was higher (SMD = 0.35; 95% CI 0.18–0.51; *I*^2^ = 72.6%; not significant Egger’s publication bias), but not significantly different.

##### Percentages of poor sleep quality pre- and during-lockdown

**FIGURE 3:**
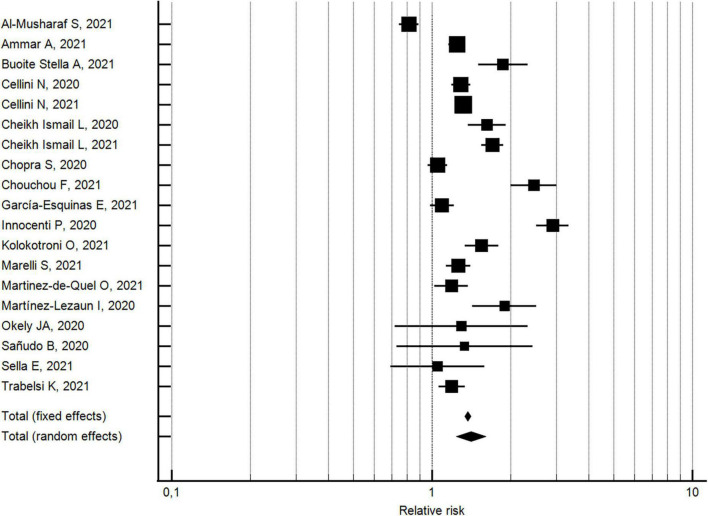
Forest plot showing pooled changes in poor sleep quality from before to during the COVID-19 lockdown. Error bars = 95% confidence interval; Square boxes = individual point estimates; and Diamond box = pooled point estimates.

The changes in the percentages of individuals with poor sleep quality from before to during the COVID-19 lockdown were evaluated considering 19 outcomes reported in 19 studies (32, 33, 40, 42, 43, 45–48, 55, 61, 63, 71–73, 76, 84, 85, 88). With respect to pre-lockdown levels, the percentage of individuals with poor sleep quality increased by around 40% during the lockdown (pooled relative risk = 1.4, 95% CI 1.24–1.61; *I*^2^ = 95.4%) (see Figure 3). The analysis did not show a significant publication bias (Egger’s *p* = 0.178).

With regard to the 13 studies (32, 40, 42, 43, 45–47, 55, 71, 72, 76, 85, 88) with a NOS quality score ≥5, the percentage of individuals with poor sleep quality increased by approximately 26% during the lockdown (pooled relative risk = 1.26, 95% CI 1.11–1.43; *I*^2^ = 936%; not significant Egger’s publication bias). With regard to the six studies (33, 48, 61, 63, 73, 84) with a NOS quality score <5, the percentage of individuals with poor sleep quality increased by approximately 83% during the lockdown (pooled relative risk = 1.8, 95% CI 1.28–2.62; *I*^2^ = 96.4%; not significant Egger’s publication bias). The two effects by risk of bias study resulted significantly different.

With regard to the 13 studies (32, 33, 42, 43, 47, 61, 63, 71–73, 84, 85, 88) using validated measurement tools, the percentage of individuals with poor sleep quality increased by approximately 32% during the lockdown (pooled relative risk = 1.3, 95% CI 1.14-1.54; *I*^2^ = 95.7%; not significant Egger’s publication bias). With regard to the six studies (40, 45, 46, 48, 55, 76) using researcher-developed tools, the percentage of individuals with poor sleep quality increased by approximately 64% during the lockdown (pooled relative risk = 1.6, 95% CI 1.28-2.10; *I*^2^ = 92.7%; not significant Egger’s publication bias). A significant difference was found for the measurement tool utilized.

##### Percentage of change in terms of improved, or worsened, or remained the same sleep quality

**FIGURE 4:**
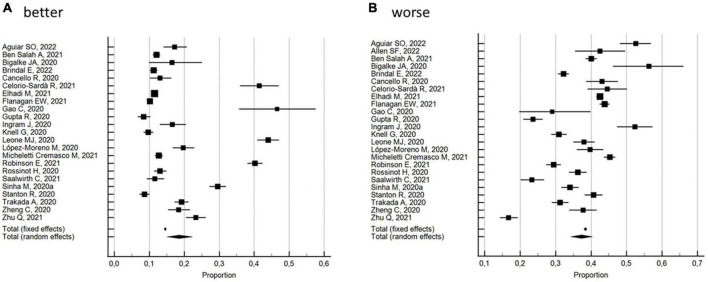
Forest plot showing pooled percentages changes in sleep quality from before to during the COVID-19 lockdown: better **(A)**, worse **(B)**. Error bars = 95% confidence interval; Square boxes = individual point estimates; and Diamond box = pooled point estimates.

The percentages of changes in individuals who improved or worsened, or maintained the same sleep quality during the lockdown with respect to pre-lockdown levels, were evaluated by 24 studies (30, 31, 36, 37, 39, 41, 44, 50, 53, 54, 57, 60, 62, 65, 67, 74, 80–82, 86, 87, 89, 91, 92). As shown in Figure 4, the random effects model yielded 57.3% (95% CI 50.01 to 61.55, *I*^2^ = 98.7%; not significant Egger’s publication bias) of participants reporting a change in sleep quality. In particular, 18.6% (95% CI 15.03–22.35; *I*^2^ = 98.9%; not significant Egger’s publication bias) reported an improvement and 37.3% (95% CI 34.27–40.39; *I*^2^ = 97.5%; not significant Egger’s publication bias) a worsening in sleep quality.

With regard to the 10 studies (30, 37, 53, 54, 57, 65, 74, 81, 89, 91) with a NOS quality score ≥5, the random effects model substantially confirmed the overall results, with 59.9% (95% CI 52.40 to 67.21, *I*^2^ = 98.7%; not significant Egger’s publication bias) of the participants reporting a change in sleep quality. In particular, 19.2% (95% CI 13.68–25.40; *I*^2^ = 98.7%; not significant Egger’s publication bias) reported an improvement and 39.0% (95% CI 34.15–44.05; *I*^2^ = 97.0%; not significant Egger’s publication bias) a worsening in sleep quality. These results were also confirmed by the 14 studies (31, 36, 39, 41, 44, 50, 60, 62, 67, 80, 82, 86, 87, 92) with a NOS quality score <5: the random effects model yielded 55.4% (95% CI 49.85 to 60.95, *I*^2^ = 98.7%; not significant Egger’s publication bias) of the participants reporting a change in sleep quality. In particular, 18.1% (95% CI 13.29–23.46; *I*^2^ = 99.1%; not significant Egger’s publication bias) reported an improvement and 36.1% (95% CI 32.11–40.26; *I*^2^ = 97.8%; not significant Egger’s publication bias) a worsening in sleep quality. Subgroup analysis by risk of bias showed significant differences.

With regard to the 2 studies (54, 65) using validated measurement tools, the random effects model yielded 80.0% (95% CI 73.86 to 85.54, *I*^2^ = 53% not heterogeneity; significant Egger’s publication bias) of the participants reporting a change in sleep quality. In particular, 44.2% (95% CI 41.18–41.27; *I*^2^ = 0% not heterogeneity; significant Egger’s publication bias) reported an improvement and 34.9% (95% CI 27.00–43.33; *I*^2^ = 63.4% not heterogeneity; significant Egger’s publication bias) a worsening in sleep quality. With regard to the 22 studies (30, 31, 36, 37, 39, 41, 44, 50, 53, 57, 60, 62, 67, 74, 80–82, 86, 87, 89, 91, 92) using researcher-developed tools, the random effects model yielded 55.2% (95% CI 51.23 to 59.13, *I*^2^ = 98.4%; not significant Egger’s publication bias) of the participants reporting a change in sleep quality. In particular, 16.6% (95% CI 13.50–19.92; *I*^2^ = 98.7%; not significant Egger’s publication bias) reported an improvement and 37.5% (95% CI 34.34–40.81; *I*^2^ = 97.7%; not significant Egger’s publication bias) a worsening in sleep quality. Subgroup analysis by measurement tool utilized showed significant differences between the percentage of change and the percentage of improvement, but not for the percentage of worsening.

#### Synthesis of sleep quality changes

Out of the three studies not included in the meta-analysis (see Table 2), 2 reported a decrease in sleep quality (38, 49) and one an increase in feelings of sleepiness during the COVID-19 lockdown with respect to pre-lockdown levels (69). This trend agrees with the results of the meta-analysis.

**TABLE 2:** Synthesis of changes in sleep outcomes from before to during the COVID-19 lockdown.

References	Synthesis by outcome		
Sleep quality			
Blume et al. (38)	Significant slight decrease in sleep quality (*p* = 0.026).		↓
Diz-Ferreira et al. (49)	Significant decrease in sleep satisfaction/sleep quality (*p* < 0.001)/half point decrease in sleep satisfaction in a 1 to 7 scale (*p* < 0.001).		↓
Majumdar et al. (69)	Increase in feelings of sleepiness (decrease in sleep quality; *p* not-reported).		↓
Sleep onset latency			
Chouchou et al. (48)	Significant increase in sleep onset latency (*p* < 0.001).		↑
Diz-Ferreira et al. (49)	Significant increase in sleep onset latency (*p* < 0.001).		↑
Elhadi et al. (50)	Significant median increase in the time it took to fall to asleep.		↑
	Increase in the percentage of participants reporting a SOL ≥30 min (from 12.90 to 25.50%) (*p* not-reported).		↑
Gupta et al. (57)	Significant increase in the percentage of participants reporting a SOL ≥30 min (from 20.50 to 43.39%, *p* < 0.001).		↑
Innocenti et al. (61)	Significant increase in the percentage of participants with a SOL >1 h (from 2.80 to 16%, *p*↑ < 0.001).		↑
Kolokotroni et al. (63)	Significant increase in sleep onset latency (*p* < 0.01).		↑
Martínez-Lezaun et al. (73)	Increase in sleep onset latency (*p* not-reported).		↑
Pépin et al. (78)	Significant increase in sleep onset latency (*p* < 0.01).		↑
Sella et al. (85)	Increase in sleep onset latency with a medium effect only in young but not in older adults.		↑Y↓O
	Slight increase in the proportion of participants with SOL ≥30 min (young: from 17.65 to 29.42%; older: from 33.30 to 38.10%		↑Y↑O
Wang et al. (90)	Approximately one third of the participants reported longer times to fall asleep.		NA
Sleep efficiency			
Cellini et al. (43)	Decrease in sleep efficiency (data and *p* not-reported).		↓
Diz-Ferreira et al. (49)	Significant decrease (*p* < 0.001).		↓
Kolokotroni et al. (63)	Significant improvement (*p*↑ < 0.01).		↑
Martínez-Lezaun et al. (73)	Decrease in the percentage of participants with a good sleep efficiency (>85%) (*p* not-reported).		↓
Ong et al. (77)	Slight but significant decrease (*p* < 0.001).		↓
Pépin et al. (78)	No significant decrease.		↓
Salehinejad et al. (83)	No significant improvement.		↑
Sella et al. (85)	No any large decrease in SE both in young and older individuals.		↓
Trabelsi et al. (88)	Significant decrease (*p* = 0.009).		↓
Sleep disturbances			
Beck et al. (35)	Among those who reported sleep problems in the previous 8 days, 54% indicated that these problems had increased since the lockdown. This was more frequently true for young people (<35 years) (60%) than for elderly people (51%, *p* = 0.02).		↑
Cheikh Ismail et al. (45)	Increase in sleep disturbances (from 52.9 to 60.8%, *p* not-reported).		↑
Cheikh Ismail et al. (46)	Increase in sleep disturbances (from 53.1 to 63.2%, *p* not-reported).		↑
Fernandez-Ballesteros and Sánchez-Izquierdo (52)	60% of the respondents did not report any changes, very few reported sleep disturbances.		=
Hetkamp et al. (58)	Increase in sleep disturbances (from 13.5 to 30%, *p* not-reported).		↑
Kolokotroni et al. (63)	Significant increase in sleep disturbances (*p* < 0.01).		↑
Mandelkorn et al. (70)	About 40% of the responders reported a worsening of sleep disturbances while a 39% reported no change.		↑
Martínez-Lezaun et al. (73)	Increase in sleep disturbances (*p* not-reported).		↑
Salehinejad et al. (83)	Significant increase (*p* < 0.001).		↑
Trabelsi et al. (88)	Significant increase (*p* < 0.001).		↑
Insomnia, sleep onset insomnia, sleep maintenance insomnia, early morning awakening insomnia			
Bacaro et al. (34)	IN	Decrease in insomnia prevalence (from 13.9 to 9.9%, *p* not-reported).	↓
Cheikh Ismail et al. (45)	SOI	Significant increase in the percentage of participants (from 19.7 to 35.4%, *p* < 0.001).	↑
	SMI	Significant increase in the percentage of participants (from 18.5 to 33%, *p* < 0.001).	↑
	EMA	Significant decrease in the percentage of participants (from 22.9 to 14.5%, *p* < 0.001).	↓
Cheikh Ismail et al. (46)	SOI	Significant increase in the percentage of participants (from 21.5 to 34.8%, *p* < 0.001).	↑
	SMI	Significant increase in the percentage of participants (from 19.1 to 25.6%, *p* < 0.001).	↑
	EMA	Significant decrease in the percentage of participants (from 23.6 to 21.5%, *p* = 0.018).	↓
Diz-Ferreira et al. (49)	IN	Significant increase in insomnia incidence (from 23.1 to 36.3%, *p* < 0.001).	↑
		Significant increase in insomnia severity score (from 17.9 to 20.8, *p* < 0.001).	↑
Gao and Scullin (54)	SMI	Significant increase in number of awakenings in the middle of the night (*p* < 0.001).	↑
		The majority of the participants reported no change in the number of night-time awakenings.	=
Ge et al. (56)	SOI	Increase in difficulty falling asleep (*p* not-reported).	↑
Hisler and Twenge (59)	SOI	Increase of participants reporting one or more days with difficulty falling asleep (from 39.1 to 76.1%, *p* not-reported).	↑
	SMI	Increase of participants reporting one or more days with difficulty staying asleep (from 41.4 to 72.7%, *p* not-reported).	↑
Innocenti et al. (61)	SMI	Significant increase in number of people with night-time awakenings or with early morning awakening (*p* < 0.0001).	↑
Kontsevaya et al. (64)	SOI	Significant increase in number of days per week with trouble falling asleep.	↑
	EMA	No significant decrease in number of days per week waking up earlier than wanted.	↓
Lin et al. (66)	IN	Increase in prevalence of clinical insomnia (from 5.7 to 9.10%, *p* not-reported).	↑
		Increase in insomnia severity (*p* not-reported).	↑
	SOI	Increase in difficulty falling asleep (*p* not-reported).	↑
	SMI	Increase in difficulty staying asleep *(p* not-reported).	↑
	EMA	Increase in waking up too early (*p* not-reported).	↑
Marelli et al. (71)	IN	Significant increase in prevalence of clinical insomnia in the students group (from 6.90 to 16.30%, *p* < 0.001) but not in the workers group (from 4.70% to 12.90, *p* not-reported).	↑S↑W
		Significant increase in insomnia severity (*p* < 0.001).	↑
	SOI	Significant increase in SOI both in students (from 38.9 to 55.4%, *p* < 0.001) and workers (from 15.1 to 41.9%, *p* < 0.001).	↑S↑W
	SMI	Significant increase in SMI both in students (from 19.5 to 33.7%, *p* < 0.001) and workers (from 24.4 to 41.9%, *p* < 0.001).	↑S↑W
	EMA	Significant increase in EMA both in students (from 21.4 to 30%, *p* < 0.001) and in workers (from 24.4 to 38.7%, *p* < 0.001).	↑S↑W
Martínez-Lezaun et al. (73)	SOI	Significant increase in trouble falling asleep during the first half hour (*p* = 0.01).	↑
	SMI	No significant increase in problems getting back to sleep after waking up during the night.	↑
Mititelu (75)	IN	Significant increase in the percentage of participants with insomnia (from 6.8 to 8.6%, *p* < 0.001).	↑
Sleep medication consumption		
Beck et al. (35)	Among those who had taken sleeping pills in the last 12 months, 41% reported taking them since the lockdown: 32% for women vs 46% for men (*p* < 0.001).	NA
Cellini et al. (42)	Decrease in the proportion of responders who used sleep medications in both Italians (from 12.3 to 10.3%, *p* not- reported) and Belgians (from 25.4 to 24.2%, *p* not-reported).	↓
Diz-Ferreira et al. (49)	No significant increase.	↑
Elhadi et al. (50)	11.3% of the participants reported they had begun to take sleep medication to help them fall asleep during the lockdown.	NA
Ge et al. (56)	Increase in sleep medications consumption (*p* not-reported).	↑
Innocenti et al. (61)	6% increase in number of people who took sleep medications 3 or more times a week, while those who did not take them decreased by about 10% (*p*↑ < 0.0001).	↑
Kolokotroni et al. (63)	Significant increase in sleep medications consumption (*p* < 0.01).	↑
Mandelkorn et al. (70)	Significant increase in sleep medications consumption in both studies (*p* < 0.001).	↑
Perez-Carbonell et al. (79)	Increase in sleep medications consumption (from 5.3 to 7.4%, *p* not-reported).	↑
Salehinejad et al. (83)	Significant increase (*p* < 0.001).	↑
Trabelsi et al. (88)	Significant increase (*p* < 0.001).	↑

Y, young; O, older; NA, not applicable; IN, insomnia: SOI, sleep onset insomnia; SMI, sleep maintenance insomnia; EMA, early morning awakening insomnia; S, students; W, workers.

### Sleep onset latency

Out of 16 studies examining sleep onset latency, 6 reported data regarding both before and during the COVID-19 lockdown and were included in the meta-analysis (43, 54, 71, 83, 88, 92); the other ten were only narratively described (48–50, 57, 61, 63, 73, 78, 85, 90).

#### Meta-analytic changes in sleep onset latency

##### Means pre- and during-lockdown

**FIGURE 5:**
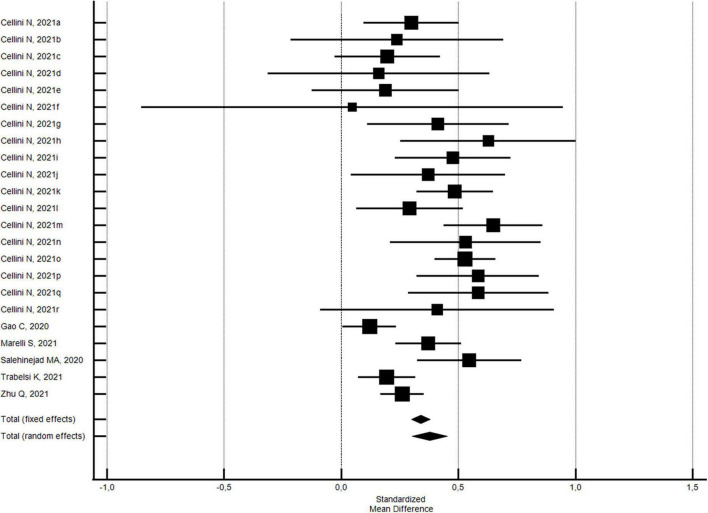
Forest plot showing pooled changes in sleep onset latency from before to during the COVID-19 lockdown. Cellini N, 2021a: Belgian Students, Female; Cellini N, 2021b: Belgian Students, Male; Cellini N, 2021c: Belgian Regular workers, Female; Cellini N, 2021d: Belgian Regular workers, Male; Cellini N, 2021e: Belgian Remote workers, Female; Cellini N, 2021f: Belgian Remote workers, Male; Cellini N, 2021g: Belgian Unemployed/retired, Female; Cellini N, 2021h: Belgian Unemployed/retired, Male; Cellini N, 2021i: Italian Students, Female; Cellini N, 2021j: Italian Students, Male; Cellini N, 2021k: Italian Regular workers, Female; Cellini N, 2021l: Italian Regular workers, Male; Cellini N, 2021m: Italian Remote workers, Female; Cellini N, 2021n: Italian Remote workers, Male; Cellini N, 2021o:Italian Stop working, Female; Cellini N, 2021p: Italian Stop working, Male; Cellini N, 2021q: Italian Unemployed/retired, Female; Cellini N, 2021r: Italian Unemployed/retired, Male. Error bars = 95% confidence interval; Square boxes = individual study effects; and Diamond box = Pooled effects.

Changes in sleep onset latency from before to during the COVID-19 lockdown were evaluated considering 23 outcomes examined by 6 studies (43, 54, 71, 83, 88, 92). The pooled results showed that there was a slightly significant increase in the sleep onset latency of a SMD of 0.38 min (95% CI 0.30–0.45; *I*^2^ = 63.7%, not shown, significant publication bias) (see Figure 5).

The results were basically the same when the NOS quality score was stratified. With regard to 21 outcomes of the four studies (43, 54, 71, 88) with a NOS quality score ≥5, the pooled results confirmed a slightly significant increase in the sleep onset latency of a SMD of 0.38 min (95% CI 0.30–0.46; *I*^2^ = 63.1%; not significant Egger’s publication bias). With regard to the outcomes of two studies (83, 92) with a NOS quality score <5, the pooled results showed that there was a slightly significant increase in the sleep onset latency of a SMD of 0.39 min (95% CI 0.11–0.66; *I*^2^ = 81.6%; significant Egger’s publication bias). No significant differences by risk of bias were found.

With regard to the 20 outcomes of the 3 studies (43, 71, 88) using validated measurement tools, the pooled results uncovered a slightly significant increase in the sleep onset latency of a SMD of 0.40 min (95% CI 0.33–0.48; *I*^2^ = 47.4%; not significant Egger’s publication bias). With regard to the outcomes of 3 studies (54, 83, 92) using researcher-developed tools, the pooled results showed that there was a slightly significant increase in the sleep onset latency of a SMD of 0.28 min (95% CI 0.10–0.47; *I*^2^ = 82.8%; not significant Egger’s publication bias). No significant differences by measurement tool utilized were found.

#### Synthesis of sleep onset latency changes

Ten studies reported 14 outcomes regarding sleep onset latency changes during the COVID-19 lockdown with respect to pre-lockdown levels: 9 (48–50, 63, 73, 78, 85, 90) reported the time it took to fall asleep and 5 (50, 57, 61, 85) the percentage of persons with a sleep onset latency ≥30 or >60 min (see Table 2).

All of the studies, with the exception of Wang et al.’s (90), reported an increase in the sleep onset latency (48–50, 57, 61, 63, 73, 78, 85). According to Sella et al.’s work (85), the increase mostly regarded young adults; Wang’s study (90) reported that approximately one third of the participants responded that it took them longer to fall asleep. Once again, this trend agrees with the results of the meta-analysis.

#### Synthesis of sleep efficiency changes

Nine studies (43, 49, 63, 73, 77, 78, 83, 85, 88) with 10 outcomes evaluated changes in the sleep efficiency during the lockdown with respect to pre-lockdown values; of these, 77.8% (7/9) reported a decrease (43, 49, 73, 77, 78, 85, 88) and 2 an increase in sleep efficiency (63, 83) (see Table 2).

#### Synthesis of sleep disturbance changes

Out of ten studies (35, 45, 46, 52, 58, 63, 70, 73, 83, 88) examining changes in sleep disturbances during the lockdown with respect to pre-lockdown levels, one study reported no change (52), and 90% (9/10) reported an increase (35, 45, 46, 58, 63, 70, 73, 83, 88) (see Table 2). According to Beck et al.’s study (35), the increase was more frequent in young with respect to older people.

#### Synthesis of the insomnia changes

Thirteen studies (34, 45, 46, 49, 54, 56, 59, 61, 64, 66, 71, 73, 75) examining 34 outcomes evaluated changes in insomnia during the lockdown with respect to pre-lockdown period: in particular, 5 evaluated changes in the insomnia status (34, 49, 66, 71, 75), 8 in sleep onset insomnia (45, 46, 56, 59, 64, 66, 71, 73), 8 in sleep maintenance insomnia (45, 46, 54, 59, 61, 66, 71, 73), and 5 in early morning awakening insomnia (45, 46, 64, 66, 71) (see Table 2). With regard to the outcomes examining the insomnia status, 88.9% (8/9) reported an increase (49, 66, 71, 75) and one decrease (34) in insomnia. Marelli et al. reported that the increase was significant in the students but not in the workers (71). All of the studies reporting on sleep onset insomnia described an increase in this variable (45, 46, 56, 59, 64, 66, 71, 73). With regard to the outcomes examining the sleep maintenance insomnia, all reported an increase (45, 46, 54, 59, 61, 66, 71, 73). Marelli et al.’s work uncovered a more marked increase in the workers with respect to the students (71). According to Gao et al. (54), the majority of the participants did not report a change in symptoms although an increase in night-time awakenings was noted. Fifty percent (3/6) of the early morning awakening insomnia outcomes described an increase (66, 71), which seemed to be more marked in the workers with respect to that in the students (71), and three a decrease (45, 46, 64).

#### Synthesis of sleep medication consumption changes

Of the eleven studies examining changes in sleep medication consumption during the COVID-19 lockdown with respect to pre-lockdown levels (35, 43, 49, 50, 56, 61, 63, 70, 79, 83, 88), 72.7% (8/11) reported an increase (49, 56, 61, 63, 70, 79, 83, 88), and one a decrease in use (43). Two studies reported that 41% (35) and 11% (50) of the participants started taking sleep medication during the lockdown (see Table 2).

## Discussion

This systematic review and meta-analysis of 63 studies aimed to examine and synthesize changes in sleep quality and sleep disturbances in the general population from before to during the COVID-19 lockdown. The main results of the meta-analysis indicated that during the lockdown, there was a significant worsening of sleep quality, and a 40% increased probability of reporting poor sleep quality. Analyses, moreover, uncovered that approximately 57% of the participants experienced a change in sleep quality, which was more frequently a worsening. Subgroup analysis showed a more marked, even if not statistically significant, worsening in sleep quality (a higher increase in the PSQI global score) and a significant higher increase in the percentage of individuals with poor sleep quality during the lockdown in the low quality studies with respect to the high quality ones, and in the studies using validated measurement tools with respect to those using researcher-developed ones. On the contrary, the percentages of participants reporting a change in sleep quality and of those experiencing a worsening are significantly higher in the high quality studies, as well as in the studies using validated measurement tools, in particular with regard to improvement. These results are partially in line with those presented by Jahrami et al. (9) who found a lower rate of sleep disturbances in the high quality studies with respect to the moderate and low quality ones, and a higher prevalence of sleep disturbances in the studies using validated measures with respect to those using researcher-developed ones (16). The meta-analysis also revealed a modest although significant increase in the sleep onset latency value (SMD = 0.38 min, 95% CI 0.30-0.45), an indicator of good sleep quality when values are lower than 30 min (21). Subgroup analysis found no significant differences by risk of bias and measurement tool utilized. Studies not included in the meta-analysis, similarly reported an increase in the time it took to fall asleep and in the percentage of individuals with a sleep onset latency ≥30 or >60. Sleep efficiency, an indicator of good sleep quality when values are ≥85% (23) also declined according to 7 out of 9 studies described in the systematic review. A growing body of evidence has demonstrated that poor sleep quality, poor sleep efficiency and longer sleep onset latency values have negative effects on health. Poor sleep quality has in fact been associated with a higher risk of type 2 diabetes (93), overweight/obesity (94), metabolic syndrome (95) and depressive and anxiety symptoms (96, 97). Similarly, a prolonged sleep onset latency has been associated with metabolic syndrome (95), and with an increased risk of cardiovascular disease (CVD) (98), and depressive symptoms (99). Finally, poor sleep efficiency is associated with a higher risk of incident CVD (100) and depression (101). Almost all of the studies included in our systematic review reported an increase in sleep disturbances, consistently with two meta-analyses that found a higher prevalence of sleep disturbances during the lockdown with respect to non-lockdown periods (9, 10). We also found an increase in the incidence, prevalence and severity of insomnia, an increase in the symptoms of sleep onset insomnia, sleep maintenance insomnia and early morning awakening insomnia. There was likewise an increase in the use of sleep medication. These results are, anew, consistent with those of other works in the literature (15, 102, 103). A significant increase in the prevalence of moderate to severe insomnia was uncovered by a study examining 900 Italian adults at the time the first rigid lockdown was implemented (102). The International COVID-19 Sleep Study, a multinational survey carried out across 14 countries, uncovered a 10% increase in a range of sleep disturbances including worse sleep quality, sleep onset and sleep maintenance problems, and greater use of hypnotics during the pandemic with respect to the precedent period. Confinement was associated with poor sleep quality and problems falling asleep (103). Finally, a recent meta-analysis found a marked increase in subthreshold insomnia symptoms but not in moderate or severe insomnia during the COVID-19 pandemic (15). Some of the long-term consequences of insomnia include impairment in cognitive performance (104), an increased risk of depression (105), and CVD (106). The lockdown did not, however, have the same effect on everyone. Indeed, our meta-analysis showed that 18.6% of individuals experienced an improvement in sleep quality, and some studies included in the systematic review reported an increase in sleep efficiency (63, 83), a decrease in insomnia prevalence (34), in early awakening sleep onset insomnia (45, 46, 64), and in sleep medication consumption (43). A variety of factors could explain these improvements in sleep parameters. According to Kocevska’s study, the lockdown’s impact on sleep largely depended on the individual’s pre-pandemic sleep quality: persons who suffered from pre-pandemic insomnia (bad sleepers) reported an improvement in sleep quality, while good sleepers before the pandemic reported a worsening in sleep quality; moreover, negative affect and worry were also found to be associated to changes in sleep quality (107). Some changes in individuals’ work routine such as the transition to working from home due to the implementation of lockdown measures may have given some the opportunity to adapt their sleep schedules to their chronotype and to sleep more, as has been underlined by some studies reporting an increased sleep duration during the pandemic (7, 8). Scarpelli et al., who found an inverse association between sleep alterations and the stringency of governmental restrictions, hypothesized that strict measures may have led some to feel safer and less vulnerable toward the virus, which translated into lower levels of anxiety and fear and better sleep (12). In accordance with that line of thought, Salanti et al. underlined that despite the general increase in the symptoms of depression and anxiety, some studies uncovered an improvement in symptoms, and he hypothesized that stricter lockdown measures may have had a positive effect on the mental health of some (17). Other psychological factors such as a highly adaptive personality and higher resilience seemed to have been associated to better sleep quality (108, 109).

The decline in sleep quality and the rise in sleep disturbances emerging from our study and the possible negative consequences of these changes on mental and physical health are cause for concern. The COVID-19 pandemic was in fact characterized by more than one wave and several sleep variables seem to have been undermined by the repeated implementation of strict restrictive measures, which may have determined an onset in sleep disturbances or aggravated them. Riva et al. pointed out that the prevalence of insomnia did not diminish when pandemic restrictions were reduced or lifted but it began to normalize only several months later and it never returned to pre-pandemic levels (102). Two studies examining the evolution of sleep variables in Italian adults during the total and partial COVID-19 lockdowns found diversified sleep patterns. Conte et al. reported that the delayed sleep timing found during the first lockdown returned to pre-pandemic levels, probably due to normalized working schedules. Instead, sleep quality, which continued to worsen during both lockdowns, appeared to be particularly sensitive to psychological variables such as stress (110). Salfi et al. reported that with regard to the period between the first and second waves of the pandemic and their relative lockdowns, on the one hand, an improvement in the symptoms of insomnia and a reduction in the prevalence of moderate/severe insomnia were noted, but on the other, the percentage of poor sleepers remained high (111).

The changes emerging in this study can be attributed to a variety of factors. The reduction in physical activity levels related to lockdown measures could have negatively affected sleep quality (112). At the same time, the greater exposure to electronic devices reported during the lockdown may have worsened sleep quality and the symptoms of insomnia, reduced sleep duration, and determined a prolonged sleep onset latency (113). Psychological factors such as an increase in loneliness (114, 115), COVID-19-related worry (82), COVID-19-related stress (116), and depressive and anxiety disorders (117, 118) may have also contributed to these changes.

Our study has several strengths. First, this systematic review and meta-analysis provided a synthesis of changes in several sleep outcomes in the general population during the lockdown, which represented an unprecedented condition within the context of a pandemic and that had a dramatic impact on daily life. Moreover, a rigorous methodological approach was used to achieve an overview of all the sleep variables linked to sleep quality and sleep disturbances *via* an extensive search of published peer-reviewed, preprint articles and gray literature, and to ensure the quality of the assessments and the synthesis of data. We summarized data using a random-effects model with a more conservative estimate to address heterogeneity given the many different ways the data were synthetized and cultural diversity across countries. There are nevertheless some limitations that must be considered when interpreting our results. The first one is represented by the high risk of bias of several of the studies included. As uncovered by subgroup analysis, the changes in sleep quality and in particular percent changes were more pronounced in the low quality studies with respect to the high quality ones. Another limitation is linked to the measurement tools because the use of non-validated instruments may have led to underestimated values. Moreover, because of the small number of the studies and the heterogeneity of the data collected, it was possible to carry out subgroup analysis only by risk of bias and measurement tool. Other limitations are linked to the characteristics of the studies included here. The majority adopted a cross-sectional design meaning that they did not collect data before the lockdown and thus did not allow to have an exhaustive evaluation of the changes in sleep outcomes. Most of the studies used convenience samples whose participants were for the most part recruited through social networks, excluding therefore individuals who are not regular internet users, such as elderly persons; this may have affected the generalizability of our results (119). Almost all of the studies utilized self-reported instruments: while on the one hand, these seem to be more sensitive to sleep disturbances (120), on the other they tend to be subject to memory recall bias and to social desirability bias. Moreover, several studies utilized a single question to collect information about very complex sleep variables. Finally, the term lockdown was frequently used to describe different degrees of restrictive measures and this too may have affected the outcomes of the study.

The findings of the present work have several research and practical implications. If on the one hand the implementation of rigid restrictive measures permitted government authorities to limit the transmission of SARS-CoV-2 infection and to mitigate its impact on health systems, on the other it has had negative consequences on sleep health in the general population. Given the importance of good sleep for our physical and mental health, sleep problems need to be detected early and treated using appropriate interventions such as the cognitive behavioral treatment for insomnia that appears to have significant effects on insomnia severity, sleep efficiency, sleep quality, sleep onset latency, and the number of awakenings (121).

As far as research is concerned, studies evaluating if and to what degree the effects on sleep health linked to the pandemic and the restrictive measures have lingered over time are warranted. These works should seek to overcome some methodological limitations by using probability-based sampling methods, validated, standardized measurement tools, and longitudinal research designs. In addition, and as underlined by other authors, instruments that are able to describe and compare lockdown measures need to be developed (122).

## Conclusion

The lockdown measures have had an important impact on sleep health in the general population. The current work uncovered a worsening in sleep quality and sleep efficiency, a prolonged sleep onset latency, and an increase in sleep disturbances and insomnia. Given the risk of chronicization of sleep disturbances and their negative impact on mental and physical health, early detection and timely interventions are crucial. High quality research based on the use of validated measurement tools and longitudinal research designs is needed to evaluate the long-term effect of lockdown measures and to identify individuals at a higher risk of developing sleep disturbances and the role of moderators.

## Author contributions

FL, CT, FP, and SM conceptualized the study. FL, PS, and CT contributed to the investigation and literature search. FL contributed to the team supervision. EP and FR performed data screening and data extraction. PS contributed to the supervision, coordination of data coding, and statistical analysis. FL and PS wrote the original draft. FL, PS, CT, MN, FC, CC, SC, ER, EP, FR, FP, and SM edited and revised critically the manuscript. All authors contributed to the article and approved the submitted version.
