# Supplementary material for: Changes in sleep quality and sleep disturbances in the general population from before to during the COVID-19 lockdown: A systematic review and meta-analysis
Source: Front Psychiatry. 2023 Apr 13;14:1166815. doi: 10.3389/fpsyt.2023.1166815 (PMC10134452; doi:10.3389/fpsyt.2023.1166815)
Supplement: Supplementary file 1 [file Table_1.pdf]

Table S1. Search Strategy: changes in sleep characteristics

**PubMed**
(sleep[MeSH Terms] OR bedtime* OR "wake time" OR waketime* OR Circadian* OR sleep OR sleeping OR insomnia OR snore OR parasomnia* OR "Life Style"[Mesh] OR lifestyle* OR “life style*”) AND (“COVID 19” OR COVID-19 OR Coronavirus OR 2019-nCoV OR “2019 nCoV” OR “SARS CoV 2” OR SARS-CoV-2 OR "COVID-19"[Mesh] OR “COVID-19 pandemic*” OR “COVID 19 pandemic*”) AND (isolation OR lock-down OR lockdown OR self-isolation OR Confinement OR Containment OR Quarantine OR "Quarantine"[Mesh])
**Web of Science**
(bedtime* OR "wake time" OR waketime* OR Circadian* OR sleep OR sleeping OR insomnia OR snore OR parasomnia* OR lifestyle* OR “life style*”) AND (“COVID 19” OR COVID-19 OR Coronavirus OR 2019-nCoV OR “2019 nCoV” OR “SARS CoV 2” OR SARS-CoV-2 OR “COVID-19 pandemic*” OR “COVID 19 pandemic*”) AND (isolation OR lock-down OR lockdown OR self-isolation OR Confinement OR Containment OR Quarantine)
**Cochrane Library**
(bedtime* OR "wake time" OR waketime* OR Circadian* OR sleep OR sleeping OR insomnia OR snore OR parasomnia* OR lifestyle* OR “life style*”) AND (“COVID 19” OR “COVID-19” OR Coronavirus OR “2019 nCoV” OR “SARS CoV 2” OR “SARS-CoV-2” OR “COVID-19 pandemic*” OR “COVID 19 pandemic*”) AND (isolation OR lock-down OR lockdown OR self-isolation OR Confinement OR Containment OR Quarantine)
**EBSCOhost**
(bedtime* OR "wake time" OR waketime* OR Circadian* OR sleep OR sleeping OR insomnia OR snore OR parasomnia* OR lifestyle* OR “life style*”) AND (“COVID 19” OR COVID-19 OR Coronavirus OR 2019-nCoV OR “2019 nCoV” OR “SARS CoV 2” OR SARS-CoV-2 OR “COVID-19 pandemic*” OR “COVID 19 pandemic*”) AND (isolation OR lock-down OR lockdown OR self-isolation OR Confinement OR Containment OR Quarantine)
**MedRxiv**[†]
bedtime bedtimes "wake time" waketime waketimes Circadian Circadians sleep sleeping insomnia snore parasomnia lifestyle lifestyles “life style” “life styles”<br>AND COVID-19 AND lockdown
**OpenGrey**
bedtime bedtimes "wake time" waketime waketimes Circadian Circadians sleep sleeping insomnia snore parasomnia lifestyle lifestyles “life style” “life styles”<br>AND coronavirus AND lockdown

[†]In Abstract or Title
