# Supplementary material for: Changes in sleep quality and sleep disturbances in the general population from before to during the COVID-19 lockdown: A systematic review and meta-analysis
Source: Front Psychiatry. 2023 Apr 13;14:1166815. doi: 10.3389/fpsyt.2023.1166815 (PMC10134452; doi:10.3389/fpsyt.2023.1166815)
Supplement: Supplementary file 2 [file Table_2.pdf]

Table S2. Risk of bias assessment

Author, year	Study design	Selection				Comparability	Outcome		Quality	
Aguiar SO, 2022	cross-sectional	c)	c)	a)	b)	a)	c)	a)	*****	5
Allen SF, 2022	cross-sectional	c)	c)	a)	b)		c)	a)	****	4
Ammar A, 2021	cross-sectional	c)	c)	b)	b)		c)	a)	***	3
Bacaro V, 2020	cross-sectional	c)	c)	a)	b)	a)b)	c)	b)	*****	5
Beck F, 2021	cross-sectional	a)	c)	b)	b)	a)b)	c)	a)	******	6
Ben Salah A, 2021	cross-sectional	c)	c)	c)	a)		c)	a)	***	3
Bigalke JA, 2020	cross-sectional	c)	c)	b)	b)	a)b)	c)	a)	*****	5
Brindal E, 2022	cross-sectional	c)	b)	b)	b)		c)	b)	**	2
Buoite Stella A, 2021	cross-sectional	c)	c)	b)	b)	a)b)	b)	a)	******	6
Cancello R, 2020	cross-sectional	c)	c)	c)	b)		c)	a)	**	2
Cellini N, 2020	cross-sectional	c)	c)	a)	b)	a)b)	c)	b)	*****	5
Celorio-Sardà R, 2021	cross-sectional	c)	c)	a)	b)		c)	b)	***	3
Cheikh Ismail L, 2020	cross-sectional	c)	c)	b)	b)	a)b)	c)	a)	*****	5
Cheikh Ismail L, 2021	cross-sectional	c)	c)	b)	b)	a)b)	c)	a)	*****	5
Chopra S, 2020	cross-sectional	c)	c)	b)	b)	a)b)	c)	a)	*****	5
Chouchou F, 2021	cross-sectional	c)	c)	b)	b)	a)	c)	a)	****	4
Diz-Ferreira E, 2021	cross-sectional	c)	c)	a)	a)	a)b)	c)	a)	*******	7
Elhadi M, 2021	cross-sectional	c)	c)	c)	a)		c)	a)	***	3
Flanagan EW, 2021	cross-sectional	c)	c)	b)	b)	a)b)	c)	a)	*****	5
Gupta R, 2020	cross-sectional	c)	c)	b)	b)	a)b)	c)	a)	*****	5
Hetkamp M, 2020	cross-sectional	c)	c)	a)	b)		c)	b)	***	3
Ingram J, 2020	cross-sectional	c)	c)	c)	a)		c)	a)	***	3
Innocenti P, 2020	cross-sectional	c)	c)	c)	b)		c)	b)	*	1
Knell G, 2020	cross-sectional	c)	c)	b)	b)		c)	a)	***	3
Kolokotroni O, 2021	cross-sectional	c)	c)	a)	b)		c)	a)	****	4
Kontsevaya AV, 2021	cross-sectional	c)	c)	a)	b)	a)b)	c)	a)	******	6
Lin LY, 2021	cross-sectional	c)	c)	a)	b)	a)	c)	a)	*****	5
López-Moreno M, 2020	cross-sectional	c)	c)	b)	b)		c)	a)	***	3
Majumdar P, 2020	cross-sectional	c)	c)	b)	b)		c)	b)	**	2
Mandelkorn U, 2021	cross-sectional	c)	c)	c)	b)	a)b)	c)	a)	****	4
Marelli S, 2021	cross-sectional	c)	c)	a)	b)	a)b)	c)	a)	******	6
Martínez-Lezaun I, 2020	cross-sectional	c)	c)	a)	b)		c)	b)	***	3
Micheletti Cremasco M, 2021	cross-sectional	c)	c)	b)	b)	a)b)	c)	a)	*****	5
Mititelu M, 2021	cross-sectional	c)	c)	b)	b)	a)	c)	a)	****	4

**Author, year**	**Study design**	**Selection**				**Comparability**	**Outcome**			**Quality**	
Perez-Carbonell L, 2020	cross-sectional	c)	c)	c)	b)		c)	a)		**	2
Robinson E, 2021	cross-sectional	c)	c)	a)	b)		c)	a)		****	4
Rossinot H, 2020	cross-sectional	b)	c)	a)	b)		c)	a)		*****	5
Saalwirth C, 2021	cross-sectional	c)	c)	a)	b)		c)	a)		****	4
Salehinejad MA, 2020	cross-sectional	c)	c)	b)	b)	a)	c)	a)		****	4
Sinha M, 2020a	cross-sectional	c)	c)	b)	b)	a)	c)	a)		****	4
Stanton R, 2020	cross-sectional	c)	c)	a)	b)		c)	a)		****	4
Trabelsi K, 2021	cross-sectional	a)	c)	b)	a)		c)	a)		*****	5
Trakada A, 2020	cross-sectional	c)	c)	b)	b)	a)b)	c)	a)		*****	5
Wang X, 2020	cross-sectional	c)	c)	b)	b)	a)	c)	a)		****	4
Zhu Q, 2021	cross-sectional	c)	c)	b)	b)		c)	a)		***	3
Blume C, 2021	cross-sectional	c)	c)	a)	b)		c)	a)		****	4
Cellini N, 2021	cross-sectional	c)	c)	b)	b)	a)b)	c)	a)		*****	5
Fernandez-Ballesteros R, 2021	cross-sectional	c)	c)	c)	b)	a)	c)	b)		**	2
Al-Musharaf S, 2021	longitudinal	c)	a)	a)	b)	a)	c)	a)	a)	*****	5
Evans S, 2021	longitudinal	c)	a)	a)	b)		c)	a)	a)	****	4
Ge F, 2021	longitudinal	c)	a)	c)	b)		c)	a)	a)	***	3
Hisler G, 2021	longitudinal	b)	b)	b)	b)	a)b)	c)	a)	d)	*****	5
Leone MJ, 2020	longitudinal	c)	a)	a)	b)	a)b)	c)	a)	a)	******	6
Maher JP, 2021	longitudinal	c)	a)	a)	b)		c)	a)	a)	****	4
Martinez-de-Quel O, 2021	longitudinal	c)	a)	b)	b)	a)b)	c)	a)	a)	******	6
Ong JL, 2021	longitudinal	a)	a)	b)	b)	a)b)	a)	a)	a)	********	8
Pépin J-L, 2021	longitudinal	c)	a)	b)	b)	a)b)	a)	a)	a)	*******	7
Sañudo B, 2020	longitudinal	c)	a)	a)	b)		c)	a)	a)	****	4
Sella E, 2021	longitudinal	c)	a)	a)	b)	a)	c)	a)	a)	*****	5
Zheng C, 2020	longitudinal	c)	a)	b)	b)	a)	c)	a)	a)	*****	5
Gao C, 2020	longitudinal	c)	c)	a)	b)	a)b)	c)	a)	a)	*****	5
García-Esquinas E, 2021	longitudinal	b)	a)	a)	b)		c)	a)	a)	*****	5
Okely JA, 2020	longitudinal	b)	a)	b)	b)		c)	a)	a)	*****	5
